# Combined Phenanthrene and Copper Pollution Imposed a Selective Pressure on the Rice Root-Associated Microbiome

**DOI:** 10.3389/fmicb.2022.888086

**Published:** 2022-05-04

**Authors:** Mingyue Li, Minmin Xu, Aoxue Su, Ying Zhang, Lili Niu, Yan Xu

**Affiliations:** ^1^College of Environmental Sciences and Engineering, Qingdao University, Qingdao, China; ^2^Shandong Academy of Environmental Sciences Co., Ltd., Jinan, China; ^3^Key Laboratory of Pollution Exposure and Health Intervention Technology, Interdisciplinary Research Academy (IRA), Zhejiang Shuren University, Hangzhou, China

**Keywords:** combined pollution, flooding condition, heavy metal, polycyclic aromatic hydrocarbon, root-associated microbiome

## Abstract

Combined organic and inorganic pollutants can greatly impact crops and microbes, but the interaction between coexisted pollutants and their effects on root-associated microbes under flooding conditions remains poorly understood. In this study, greenhouse experiments were conducted to investigate the individual and combined effects of phenanthrene (PHE) and copper (Cu) on rice uptake and root-associated microbial coping strategies. The results showed that more than 90% of phenanthrene was degraded, while the existence of Cu significantly reduced the dissipation of PHE in the rhizosphere, and the coexistence of phenanthrene and copper promoted their respective accumulation in plant roots. Copper played a dominant role in the interaction between these two chemicals. Microbes that can tolerate heavy metals and degrade PAHs, e.g., *Herbaspirillum*, Sphingobacteriales, and Saccharimonadales, were enriched in the contaminated soils. Additionally, microbes associated with redox processes reacted differently under polluted treatments. Fe reducers increased in Cu-treated soils, while sulfate reducers and methanogens were considerably inhibited under polluted treatments. In total, our results uncover the combined effect of heavy metals and polycyclic aromatic hydrocarbons on the assemblage of root-associated microbial communities in anaerobic environments and provide useful information for the selection of effective root-associated microbiomes to improve the resistance of common crops in contaminated sites.

**Graphical Abstract:**
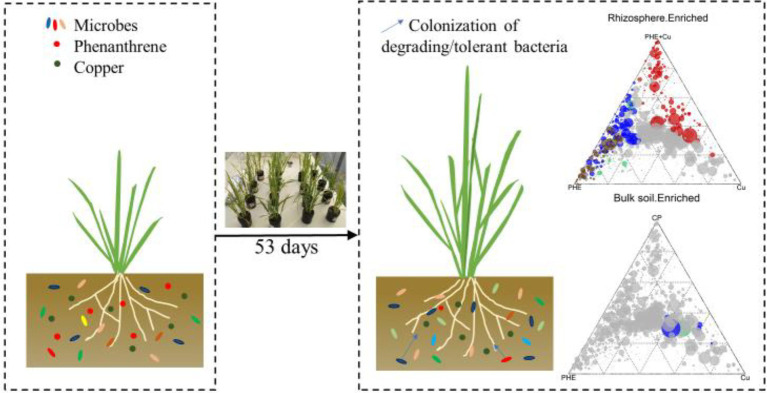


## Introduction

Soil pollution has become increasingly serious due to the development of socially productive forces (Zhang and Chen, 2017). The toxic effects and remediation of heavy metals or polycyclic aromatic hydrocarbon (PAH) pollution in soils have been extensively studied (Cristaldi et al., 2017). However, as the coexistence of these two pollutants widely occurs in contaminated soils, the synergetic effects produced by the interaction between heavy metals and PAHs could pose a more serious threat to agricultural ecological safety (Chigbo et al., 2013). For example, when Cu^2+^ was combined with 1,2-dihydroxyanthraquinone (1,2-dhATQ), an increased inhibition effect occurred on plant photosynthesis compared to 1,2-dhATQ alone (Babu et al., 2001). Plants growing in contaminated soils can not only accumulate pollutants in their bodies but also affect the degradation and transformation of pollutants by regulating their surrounding rhizosphere microbiome (Marchal et al., 2014; Luo et al., 2017). However, previous studies mainly focused on the effects of pollutants on plant growth and changes in soil microbial communities (Alkio et al., 2005; Zhang et al., 2011; Lu et al., 2013). The regulatory strategies of plants themselves on their root-associated microbiome to cope with pollution stress are still limited.

Typically, microbes have strong remediation and stabilization abilities for polycyclic aromatic hydrocarbons and heavy metals (Cao et al., 2007). The form or bioavailability of pollutants can be altered by microbial metabolism and thus effectively improve the degradation of organic pollutants and the transformation of heavy metals (Teng and Chen, 2019; Li et al., 2021a). As the key interface between plants and microorganisms, the root system is of great importance in studying the coping strategies of the root-associated microbiome and plants themselves to counter contamination stress (Liu et al., 2020a). Generally, the root-associated microbiome includes endophytes that colonize the interior roots and microbes that colonize around the roots (Sloan and Lebeis, 2015). Root microorganisms can help plants improve the utilization of nutrients in the environment and cope with diseases, pests, and abiotic stresses (Trivedi et al., 2020). Meanwhile, plants can regulate the composition of microbes in the root system and select specific microbes that are favorable for colonization. For example, a previous study showed that rice plants could change their rhizosphere microenvironment and nitrogen utilization efficiency by recruiting nitrogen-transforming microbes that survive in the root system (Zhang et al., 2019). With the combined contamination of phenanthrene, n-octadecane, and cadmium, legumes could select functional microbial communities to colonize the rhizosphere, and moreover, various legume species have the potential to construct different combinations of microbial species (Jiao et al., 2019). This means that separate factors, such as plant genotypes and soil types, could contribute to distinct root-associated microbial compositions (Berendsen et al., 2012). Therefore, it is of significance to study classical plants that cooperate with their root microbiome to cope with pollution stress under a specific environment. However, previous studies focused on the hyperaccumulating plants used for pollution remediation (Lucisine et al., 2014; Visioli et al., 2014), but the research on the assemblage of root-associated microbiomes by typical crops and the response strategies of microbial communities under pollution stress still need to be addressed.

Compared to single contaminants, it is necessary to study the interaction mechanism between heavy metals and PAHs in the soil microbial communities and the growth of plants due to their universality in contaminated sites. A combination of heavy metals and PAHs may have a synergetic effect on plant growth (Maliszewska-Kordybach and Smreczak, 2003; Zhang et al., 2012). Our previous study on wheat plants reported that when heavy metals and PAHs existed simultaneously, the mutual influence increased their accumulation in the interior roots (Xu et al., 2021). This is different from the findings in Chen et al. (2020) that copper reduced the accumulation of PAHs in spinach roots and stems. Furthermore, the coexistence of Cd and pyrene significantly inhibited microbial biomass and respiration in red earth rice soils (Lu et al., 2013). These indicate that the effects of both heavy metals and PAHs on plants and microbial communities may be associated with pollutant types, plant species, and environmental factors (Lu et al., 2013; Chen et al., 2020; Xu et al., 2021). Usually, the biodegradation of PAHs and heavy metals could be enhanced in aerobic environments (Chang et al., 2006). The phenanthrene-degrading bacteria previously discovered are mostly aerobic bacteria, such as the aerobic heterotrophic bacterium *Sphingomonas*, which is an effective phenanthrene-degrading bacterium and has good copper resistance (Song et al., 2017). However, PAHs can also be quickly degraded under anaerobic conditions, and the interaction between heavy metals and PAHs has synergistic effects on microbial respiration and microbial biomass under anaerobic conditions (Babu et al., 2001; Deng et al., 2018). There are differences in metabolic pathways and microbial community structures under separate oxygen conditions in combined contaminated soils (Teng and Chen, 2019; Thomas et al., 2019). Hence, it is worth studying the interaction between PAHs and heavy metals and the strategies of hygrophilous plants, as well as the microbiome in response to pollution stress under anaerobic conditions to unravel whether there are differences between anaerobic and aerobic environments.

To explore the effects of combined pollutants of both PAHs and heavy metals on the root-associated microbiome in flooded paddy soils and the strategies of crops and root-associated microbes in dealing with combined pollution, a greenhouse experiment using classic crop rice cultivar (*Oryza sativa L.*) has been conducted. Phenanthrene (PHE), a typical representative of PAHs, and heavy metal copper (Cu) were chosen as the model compounds to study: (1) the degradation dynamics of PHE with the coexistence of Cu under the condition of rice planting; (2) the effect of combined PHE and Cu pollution on the assemblage of root-associated microbial community clustering processes; and (3) strategies for plant regulation of root-associated microbiome to cope with combined contamination.

## Materials and Methods

### Contaminated Soil Preparation

Top soils (0–20 cm) were taken from a paddy field in Shaoxing, Zhejiang Province, China (29° 14′ 12″ N, 119° 53′ 49″E), and all the soils were from the same plot. The soil was naturally air-dried and sieved with a 2-mm sieve before use. The soil geochemical properties were determined as follows: soil dissolved organic carbon (DOC) was assayed using a spectrophotometer (L4, YouKe, Shanghai, China) followed by colorimetric determination (Zhan and Zhou, 2002). An inductively coupled plasma-optical emission spectrometer (ICP-OES, iCAP 6,300 DUO, Thermo Fisher Scientific Inc., Waltham, MA) was used to assay the content of nutrient elements after digestion in a mixture of HNO_3_: HF: HClO_4_ (ratio of 2:2:1; Ge et al., 2021). Soil pH was assayed using a pH meter (PHS-3C, Leica, Shanghai) with soil-to-water ratio of 1:2.5. Detailed data of soil properties are provided in Supporting Information (Supplementary Table S1).

The original soils were divided equally into four parts. For copper-treated soil, 211 mg of CuCl_2_ was dissolved in 50 ml sterilized water and then added to 1 kg soil to make the concentration of copper in the soil at 100 ppm. For PHE-treated soil, 50 ml phenanthrene solution (2 g L^−1^ dissolved in acetone) was added to 1 kg soil. The original soil has been detected the copper and phenanthrene with concentrations of 43.46 and 0 mg kg^−1^ dry soil, respectively, and finally, the measured concentrations of Cu and PHE in the contaminated soils were 152.73 and 45.56 mg kg^−1^ dry soil, respectively. Particularly, to meet the needs of N, P, and K during plant growth, the tested soils were fertilized with a mixture of CH_4_N_2_O (0.32 g kg^−1^), KH_2_PO_4_ (0.66 g kg^−1^), and KCl (0.29 g kg^−1^). The added concentration of Cl^−^ in our study was ranged from 137.9 to 179.5 mg kg^−1^. After thoroughly mixing, the skipped soils were aged for 1 week in the dark at room temperature before use.

### Experimental Design and Sampling

To analyze the effects of Cu, PHE and their combined existence on the rice root-associated microbiome, a greenhouse experiment was designed with eight treatments as follows: (a) phenanthrene (PHE) with and without plants; (b) copper (Cu) with and without plants; (c) phenanthrene and copper (CP) with and without plants; and (d) unpolluted (control) with and without plants. Each treatment was conducted in four replicates. Yongyou 12 (*hybrid*) was used as the tested rice plant. The rice seeds germinated on a sterilized petri dish, and after germination, the seedlings were transferred to a Hoagland nutrient solution to grow for 2 weeks. Then, uniform plants were selected and transplanted into the four treated soils mentioned above. Two hundred grams of soil was contained in a black polyvinylchloride pot, and two plants were incubated in one pot. During the planting period, keep the incubated soil flooded with a 2–3 cm sterilized water layer consistently. After 53 days of growth, the plant roots had fully filled the wall, and then, all the plants and soils were collected for analysis.

The collection of endosphere, rhizosphere, and bulk soil was conducted according to Edwards et al. (2015). Briefly, after plants were totally removed from the pots, loosely bound soil in the roots was gently removed by hand, and 1 mm of soil attached to the root was left. Then, the roots were soaked in PBS buffer solution and stirred to disperse the rhizosphere soil into the buffer solution. Then, the rinsed root was placed in a new PBS buffer solution, sonicated intermittently for 1 min, and rinsed twice with sterile water. The rinsed roots were collected as endosphere compartments. In addition, the unplanted soil was stored as a bulk soil compartment. Finally, all the soil and root samples were frozen at −80°C for further determination.

### DNA Extraction, Amplification, and 16S rRNA Gene Sequencing

The genomic DNA of roots and soils was extracted using the FastDNA SPIN kit (MP Biomedicals, United States) according to the instructions of the kit (Liu et al., 2020b). The concentration and purity of the DNA were detected by a NanoDrop One spectrophotometer (Thermo Fisher Scientific, MA, United States; Di et al., 2020). Using genomic DNA as a template, the primer pair 799F and 1193R was used for bacterial PCR amplification, and the Arch340F, Arch1000F and Uni519F, Ach806R primers were used for archaeal nested PCR amplification. Barcodes were ligated to the forward primer to distinguish between samples. According to the principle of equal mass, the required volume of each sample was calculated, and then, PCR products were mixed thoroughly. Finally, PCR amplicons were purified and sequenced on an Illumina Nova6000 platform (Guangdong Magigene Biotechnology Co., Ltd. Guangzhou, China). The sequences are available in NCBI Sequence Read Archive (SRA) database (accession number is SRP348161).

The quality of raw data was controlled by Fastp (version 0.14.1).^1^ UPARSE (UPARSE v.7.0.1001) was applied to cluster the qualified sequences into operational taxonomic units (OTUs) at 97% identity. The Silva^2^ database was used to annotate the taxonomic information by Usearch-Sintax (set the confidence threshold to default to ≥0.8).

### Chemical Analysis of Sampled Soils and Plants

The soil and plants were freeze-dried using a freeze drier (CTFD-10S, Chuangxin Electronic Technology Co. Ltd., Qingdao Yonghe). The determination of PHE was conducted following the previous study (Szulejko et al., 2014). Briefly, freeze-dried roots and soils were extracted three times with a mixture of acetone and hexane (1:1) by ultrasonication for 30 min each time. The supernatant was centrifuged and transferred to a new glass bottle and then concentrated by nitrogen blowers and redissolved with 2 ml of *n*-hexane. A gas chromatograph (GC; Agilent 7890A, Agilent, Santa Clara, CA, United States) equipped with an HP-5 MS capillary column (30 m by 0.32 mm diameter by 0.25 mm; J&W Scientific, Inc., Folsom, CA, United States) was used for the quantification. The instrumental program was set as follows: the initial temperature was 70°C, maintained for 2 min, increased at 15°C·min^−1^ to 280°C and then held constant for 3 min. The concentrations of total and available Cu were assayed using an inductively coupled plasma-optical emission spectrometer (ICP-OES, iCAP 6,300 DUO, Thermo Fisher Scientific Inc., Waltham, MA). For total copper, the freeze-dried soils and plants were digested in a mix of HNO_3_/HF/HClO_4_ (volume ratio of 2:2:1), and the digestion liquid was then dissolved in 25 ml of distilled water before detection (Ge et al., 2021). The available Cu in soil was extracted with a DTPA mixture (0.005 moll^−1^ DTPA, 0.01 mol L^−1^ CaCl_2_, 0.1 mol L^−1^ TEA). Briefly, 2 g of dried soil was added to 5 ml DTPA mixture and then shaken at 20°C for 2 h (120 rpm), after which the mixture was centrifuged at 12000 *g* for 10 min and filtered through a 0.22-*μ*m membrane before measurement.

Quality control guidelines were followed throughout the analysis process. The recoveries of phenanthrene and copper are 86.8–89.2% and 94.6–104%, respectively. The external standard method was used, and the calibration R^2^ of the generated standard regression curves for phenanthrene and copper ranged from 0.98 to 0.99. The performance of the instrument was tested with solvent blanks per 12 samples. In addition, three replicates were used for all analyses.

### Statistical Analysis

All figures and data analysis were performed using Origin2018 and R software (x64 4.0.3). Microbial *α*-diversity and community composition were analyzed from an online analysis platform (Guangdong Magigene Biotechnology Co., Ltd.).^3^ The β-diversity of the microbial community was represented by principal coordinate analyses (PCoAs) using Bray–Curtis distances. The significance of microbial communities among separate treatments and rhizocompartments was tested with permutational multivariate analysis of variance (PERMANOVA) using the *vegan* package in R. To clearly demonstrate the influence of Cu, PHE, and Cu + PHE on the rice root-associated microbiome, the *EdgeR* package was used to select the significantly enriched and depleted OTUs between polluted and unpolluted rhizocompartments (endosphere, rhizosphere, bulk soil). Multiple hypothesis testing was used to correct *p* values, and a value of 0.05 (*adj.* value of *p* ≤ 0.05) was chosen as the threshold for statistical significance. Differentially significant OTUs were expressed by ternary plots using the *grid* package in R.

## Results and Discussion

### Residual Concentrations of Phenanthrene and Cu in Rice Roots and Soils

**Figure 1:**
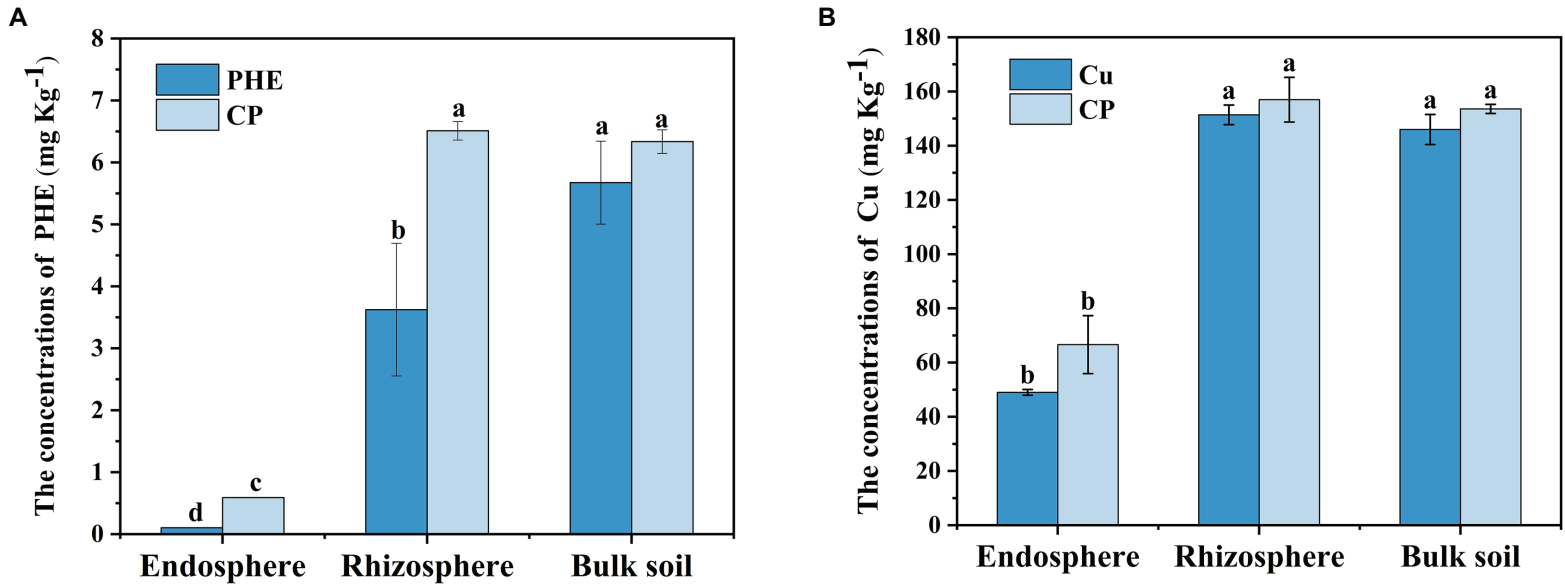
The residual concentrations of phenanthrene **(A)** and Cu **(B)** in different rhizocompartments. Symbol meaning: PHE, phenanthrene-only treatment; Cu, Cu-only treatment; CP, composite treatment of phenanthrene and Cu. Different letters indicate treatment differences (*p* < 0.05).

The residual concentrations of PHE and copper in different treatments and rhizocompartments were detected (Figure 1). Following 53 days of growth, most of the PHE was degraded, with the residual concentrations of 3.63–6.51 mg kg^−1^ in the rhizosphere and unplanted bulk soil (Figure 1A). Notably, the degradation rate of PHE in rhizosphere soil (an average of 92.0%) was significantly (*p* < 0.05) higher than that in unplanted bulk soil (an average of 87.6%), which indicated that plants played a positive role in the PHE dissipation process. Similar results have been found in a previous study, in which plants can improve the bioavailability and degradation rate of PAHs by increasing root exudates and stimulating the rhizosphere microorganisms (Cheema et al., 2009). We noted that the residual concentration of PHE was higher in the combined PHE + Cu treatment group than that in the single PHE treatment group. This may be due to the inhibited activities of the functional rhizosphere microbiota and plants by Cu stress, which further weakened the degradation of phenanthrene in the rhizosphere. In the unplanted bulk soil, the weakening effect induced by Cu was not clear, which further confirmed that the Cu inhibition effect on PHE degradation was mainly plant-related. Nevertheless, the interactions between heavy metals and organic matter in the soil, such as complexation and precipitation effect, could also significantly influence the degradation of PHE, as the previous study noticed (Chen et al., 2020). In the endosphere, the concentrations of PHE were approximately 0.10 and 0.59 mg kg^−1^ under single PHE and PHE + Cu combined treatment groups, respectively. The synthetic effect of PHE and Cu increased the enrichment of PHE in plant roots. Heavy metal ions can change the permeability of the soluble part of the plasma membrane in plants, making the membrane body fragile and heavy metals easier to enter, which promotes the entry of PAHs into rice roots (Boojar and Goodarzi, 2007).

In the case of the Cu-treated group, compared with the initial concentration (152.73 mg kg^−1^), the Cu content in the rhizosphere and bulk soil under single Cu treatment decreased slightly, with the concentration ranging from 146.02 to 151.40 mg kg^−1^, respectively. However, the Cu concentration was higher under combined pollution treatments, with the concentration ranging from 153.09 to 157.03 mg kg^−1^ in the rhizosphere and bulk soil, respectively (Figure 1B). The decrease in extracted Cu was likely due to the adsorption of Cu by minerals or organic substances in the soil environment (Misra and Pandey, 2004; Dong et al., 2020). There was no significant change in Cu concentration between the rhizosphere and bulk soil or between the Cu-only treatment and PHE + Cu treatment groups, implying a weak impact of plants and PHE on Cu accumulation. In contrast, the concentration of effective Cu in the rhizosphere was significantly higher than that in bulk soil (Supplementary Figure S1), which might be due to the activation of plant roots by the release of root exudates (van der Ent et al., 2016). In addition, Cu was assimilated to the interior rice roots (ranging from 48.28 mg kg^−1^ to 74.18 mg kg^−1^), and phenanthrene had no significant effect on the accumulation of copper in rice roots. This is consistent with previous studies (Xu et al., 2021). Plants can increase the availability of heavy metals by releasing organic matter and chelating compounds, which increased the active copper in our experiment (Supplementary Figure S1). In general, the combined PHE + Cu pollution considerably promoted the accumulation of PHE and Cu in rice interior roots, and the heavy metal Cu reduced the degradation rate of PHE in the rhizosphere soil.

### Root-Associated Bacterial Community Composition and Diversity Among Different Treatments

**Figure 2:**
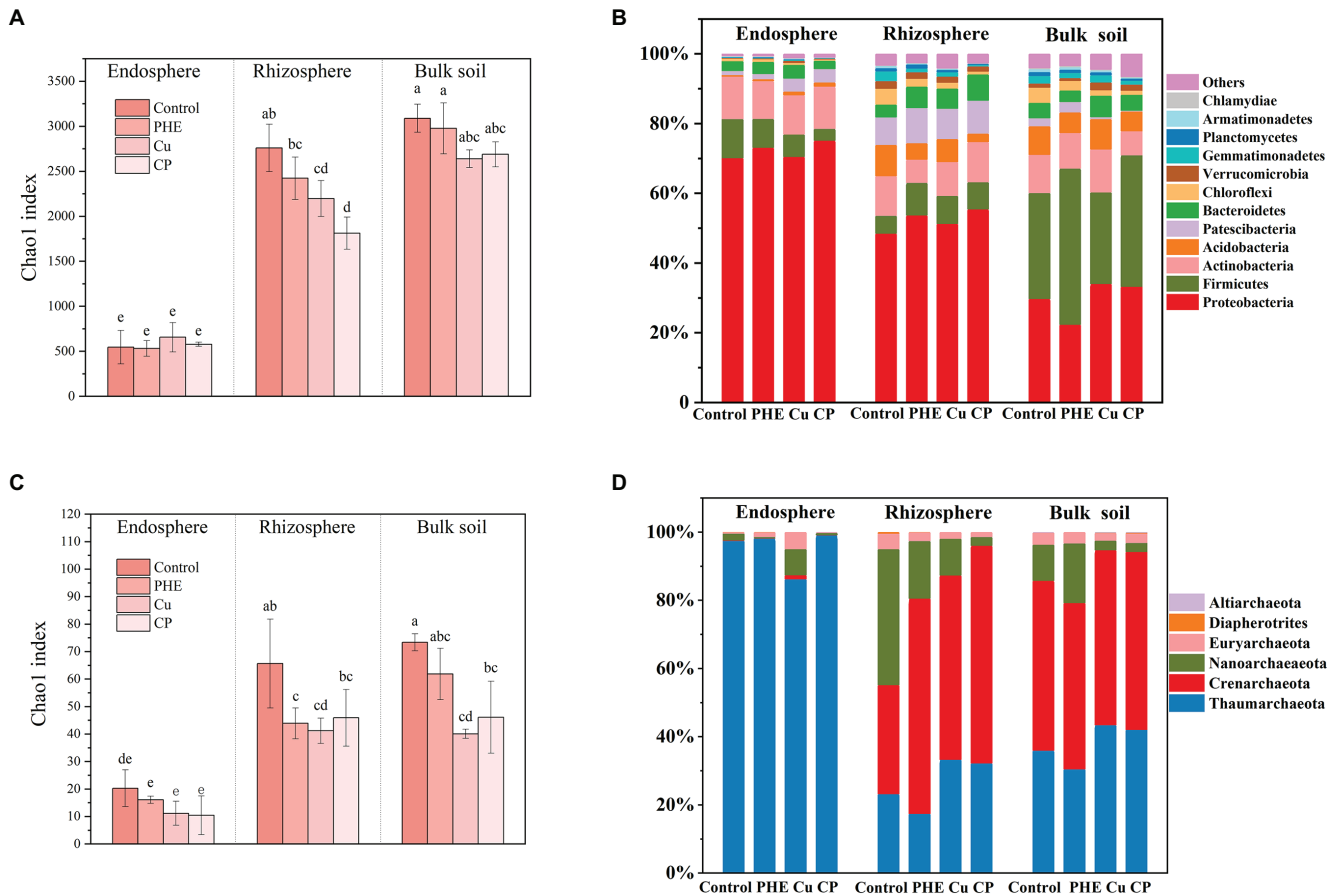
Chao 1 index **(A**,**C)** and microbial community composition **(B**,**D)** of bacteria **(A**,**B)** and archaea **(C**,**D)**. The name abbreviation of treatment is the same as Figure 1. Different letters indicate treatment differences (*p* < 0.05).

Figure 2 shows the *α*-diversity and composition of bacterial and archaeal communities. In the original soil, the Chao1 index was 3089.47 with the microbial communities were mainly composed of Firmicutes (30.4%), Proteobacteria (29.7%), Actinobacteria (11.0%), Acidobacteria (8.2%), Bacteroidetes (4.4%), Chloroflexi (4.3%), and others (12.0%). Microbial *α*-diversity showed that the diversity of bacterial communities in the endosphere was significantly lower than that in both rhizosphere and bulk soil, which was due to the strong screening effect of plant roots on endophytic microbial colonization (Figure 2A), as most previous studies mentioned (Sun et al., 2021; Xu et al., 2021). Additionally, the addition of Cu and/or PHE reduced the microbial community richness to some extent, and the influencing effects on each rhizocompartment were significantly different. Overall, the influence of pollutants on rhizosphere bacteria was greater than that in the bulk soil, and the influence on the endosphere was the least noticeable (Figures 2A,B). Both PHE, Cu and Cu + PHE treatments had no significant effects on the bacterial community richness in the endosphere. In the bulk soil, the Chao1 index of bacterial communities under both unpolluted control and PHE-only treatments was relatively higher than those under both Cu-only and combined Cu + PHE treatments, but the difference was not significant. In the rhizosphere, both the Cu-only and combined Cu + PHE treatments had a significantly (*p* < 0.05) stronger impact on bacterial *α*-diversity than the PHE-only treatment, implying that copper stress has a stronger negative effect on rice root-associated bacterial community diversity than phenanthrene. This might be because most of the phenanthrene has been degraded and absorbed in our tested soil (Figure 1A), while the heavy metal Cu is more stable and can exist for a long time. Notably, we also found that the coexistence of PHE and Cu had a stronger effect on rhizosphere bacterial community diversity than that of single contamination. On the one hand, this may be due to the enhanced toxic effect of combined pollutants on plant growth, which might weaken the protective effect of plants on associated rhizosphere microbes (Babu et al., 2001). Similarly, previous research studied the interaction of Cu and pyrene on the phytoremediation potential of *B. juncea* and found that the uniform effect of compound pollution on the growth of *B. juncea* was greater than that of single pollution (Chigbo et al., 2013). On the other hand, combined pollutants might have more toxic effects on some microbes than single pollutants, leading to significantly low microbial diversity in the rhizosphere. Lu et al. (2013) found that the biological toxicity of pyrene combined with cadmium to soil microbial communities was significantly greater than that of cadmium or pyrene alone, which is consistent with our findings.

**Figure 3:**
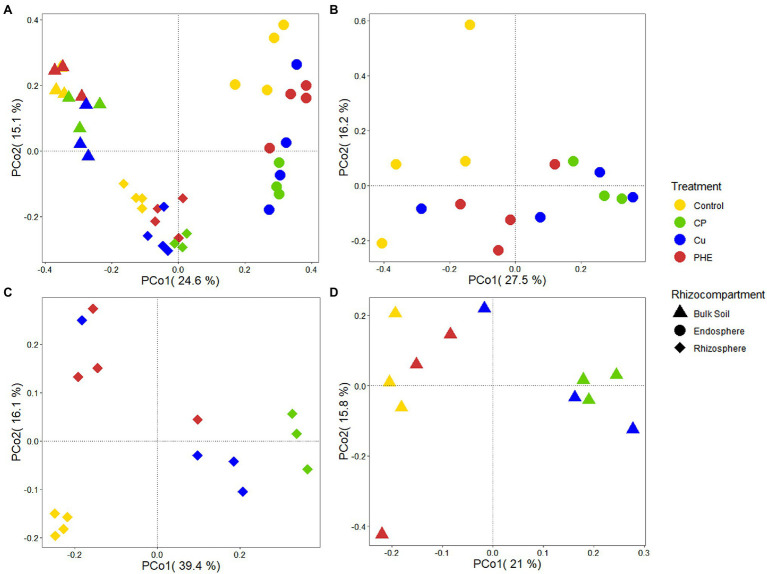
PCoA analysis of whole bacterial communities **(A)**, as well as the separate bacterial communities of endosphere **(B)**, rhizosphere **(C)** and bulk soil **(D)** under different treatments. The name abbreviation of treatment is the same as Figure 1.

Microbial community structure and diversity analysis under several root zones and treatments were performed to evaluate the effects of contaminants and rhizocompartments on the root-associated bacterial ecology of rice (Figures 2, 3). The endophytic bacteria of the tested rice plants mainly consisted of Proteobacteria, Actinobacteria, and Firmicutes (Figure 2B), which was similar to previous studies found in rice and *Arabidopsis* (Edwards et al., 2015). Additionally, we found that the relative abundance of Proteobacteria in the endosphere accounted for more than 70%, which was significantly higher than that in rhizosphere and bulk soil. Compared to the rhizosphere, the relative abundances of most bacterial phyla, e.g., Acidobacteria, Gemmatimonadetes, Verrucomicrobia, and Planctomycetes, dramatically decreased in the endosphere, which further confirmed that plants can actively select microbes to colonize their interior roots. Furthermore, microbes in the relative abundance of Proteobacteria, Actinobacteria, and Verrucomicrobia were greater in the rhizosphere soil than in the bulk soil (Figure 2B). This is because the growth of bacteria is regionally selective, and plants can provide favorable growth conditions for these selected species by secreting root exudates (Hu et al., 2018), thus recruiting specific microorganisms to colonize the rhizosphere microenvironment (Rodriguez et al., 2019). Considering the influence of pollution on the bacterial composition, the changes in the relative abundance of Firmicutes and Proteobacteria were almost the largest. These two phyla have strong adaptability and are the dominant microbes in many soils under heavy metal stress (Drzewiecka, 2016) and play an important role in the degradation of PAHs (Sun et al., 2010; Li et al., 2021b). Furthermore, principal coordinate analyses (PCoA) on Bray–Curtis distances were performed to investigate the variation in microbial communities in different rhizocompartments under separate treatments (Figure 3). Along the X-axis, the microbial diversity was clearly distinguished according to the rhizocompartment, and the bacterial distribution was visibly separated from the endosphere and the rhizosphere to the unplanted bulk soil. Along the Y-axis, the microbial communities were apparently differentiated by the polluted treatments. The distributive distance of the points representing the PHE, Cu, and PHE + Cu treatments increased successively compared to that of the unpolluted control group, which was consistent with our microbial α-diversity analysis. This indicates that the effects of PHE, Cu, and combined pollution on the bacterial community increased sequentially. Permutational multivariate analysis of variance (PERMANOVA) results further confirmed the significant influence of treatments and rhizocompartments on the bacterial communities (Supplementary Table S2). The effect of rhizocompartments on bacterial communities (*R*^2^ = 0.355, *p* < 0.001) was greater than that of pollution treatments (*R*^2^ = 0.128, *p* < 0.001). This suggests that rhizocompartments constituted the largest source of bacterial community variability (Figure 3A), which was consistent with previous studies (Edwards et al., 2015). The type of contaminant constitutes a secondary source of bacterial diversity, although it did not alter the separation pattern of rhizosphere compartments. Additionally, the bacterial community variation in separate rhizocompartments was also analyzed using both PCoA and PERMANOVA to further evaluate the effects of Cu and/or PHE pollution on the bacterial communities (Figures 3B–D). Our results showed that the effect of pollution treatments on the bacterial communities was the most in the rhizosphere (*R*^2^ = 0.513, *p* < 0.001), followed by that in the endosphere (*R*^2^ = 0.338, *p* < 0.001). The pollution effect on the microbial communities in the unplanted bulk soil was the least significant (*R*^2^ = 0.335, *p* = 0.032). This may be related to the plant shaping effect on the root-associated microbial community structure and the screening of endophytes (Xiong et al., 2021).

### Root-Associated Archaeal Community Composition and Diversity Among Different Treatments

**Figure 4:**
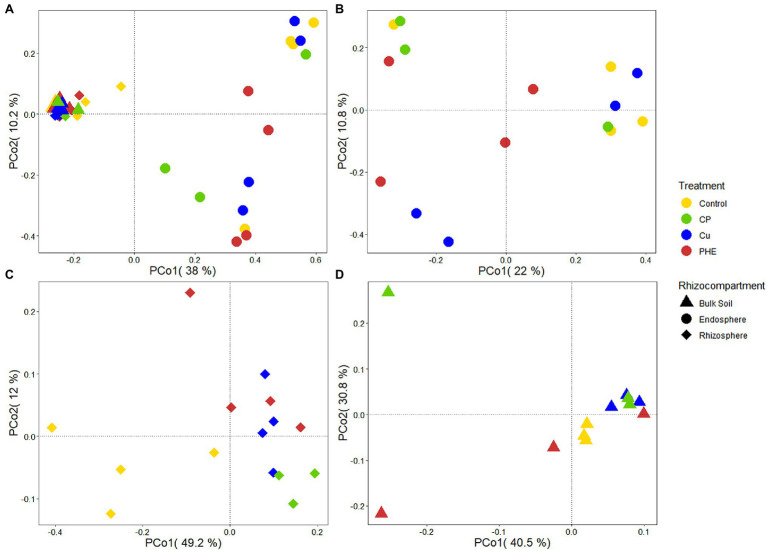
PCoA analysis of whole archaeal communities **(A)**, as well as the separate archaeal communities of endosphere **(B)**, rhizosphere **(C)**, and bulk soil **(D)** under different treatments. The name abbreviation of treatment is the same as Figure 1.

Similarly, the archaeal community variation among different rhizocompartments and treatments was also analyzed to fully evaluate the exogenous pollution on root-associated microbial ecology (Figures 2, 4). The archaeal communities were mainly composed of Thaumarchaeota, Crenarchaeota, Nanoarchaeaeota, and Euryarchaeota in both rhizosphere and unplanted bulk soils (Figure 2D). In the endosphere, the dominant archaeal group was Thaumarchaeota, with a proportion of 97.5–99.0% in all treatments. Moreover, it seems that archaeal diversity was more easily impacted by exogenous contamination than bacteria (Figure 2D). Similar to bacteria, the *α*-diversity of the archaeal community in the endosphere was significantly lower than that in the rhizosphere and unplanted bulk soil. However, the addition of pollutants significantly reduced the richness of archaeal communities (Figure 2D). Except for both the Cu-only and combined Cu + PHE treatments, the PHE-only treatment also had a significant negative influence on archaeal *α*-diversity and composition in the rhizosphere, implying that archaeal communities were susceptible to phenanthrene.

It can be seen from the PCoA analysis of archaeal communities that along the X-axis, archaeal communities are mainly divided into two parts: the endosphere part and the rhizosphere combined unplanted bulk soil part (Figure 4A). The dispersion degree of microbial communities in the rhizosphere and bulk soil was much weaker than that in the endosphere. According to the result of PERMANOVA (Supplementary Table S3), there was a significant difference in the archaeal communities caused by rhizocompartments (*R*^2^ = 0.373, *p* < 0.001) but no significant difference caused by pollution treatments on the whole archaeal communities (*R*^2^ = 0.640, *p* = 0.130). Similar to the bacteria, the rhizocompartment also constituted the main source of microbial community diversity. It seems that this pattern is similar in different plants, and previous studies on tomatoes have also found the significant differences in archaeal communities in different rhizocompartments (Lee et al., 2019). Separately, PHE and/or Cu pollution had significant effects on the rhizosphere archaeal communities (*R*^2^ = 0.492, *p* = 0.004; Figures 4B–D; Supplementary Table S3). Although the PCoA in the endosphere was more discrete, the effect of different treatments on the archaeal community in the endosphere was not significant (*R*^2^ = 0.261, *p* = 0.56; Figure 4D; Supplementary Table S3).

### Significantly Different Bacterial OTUs Among Polluted Treatments

**Figure 5:**
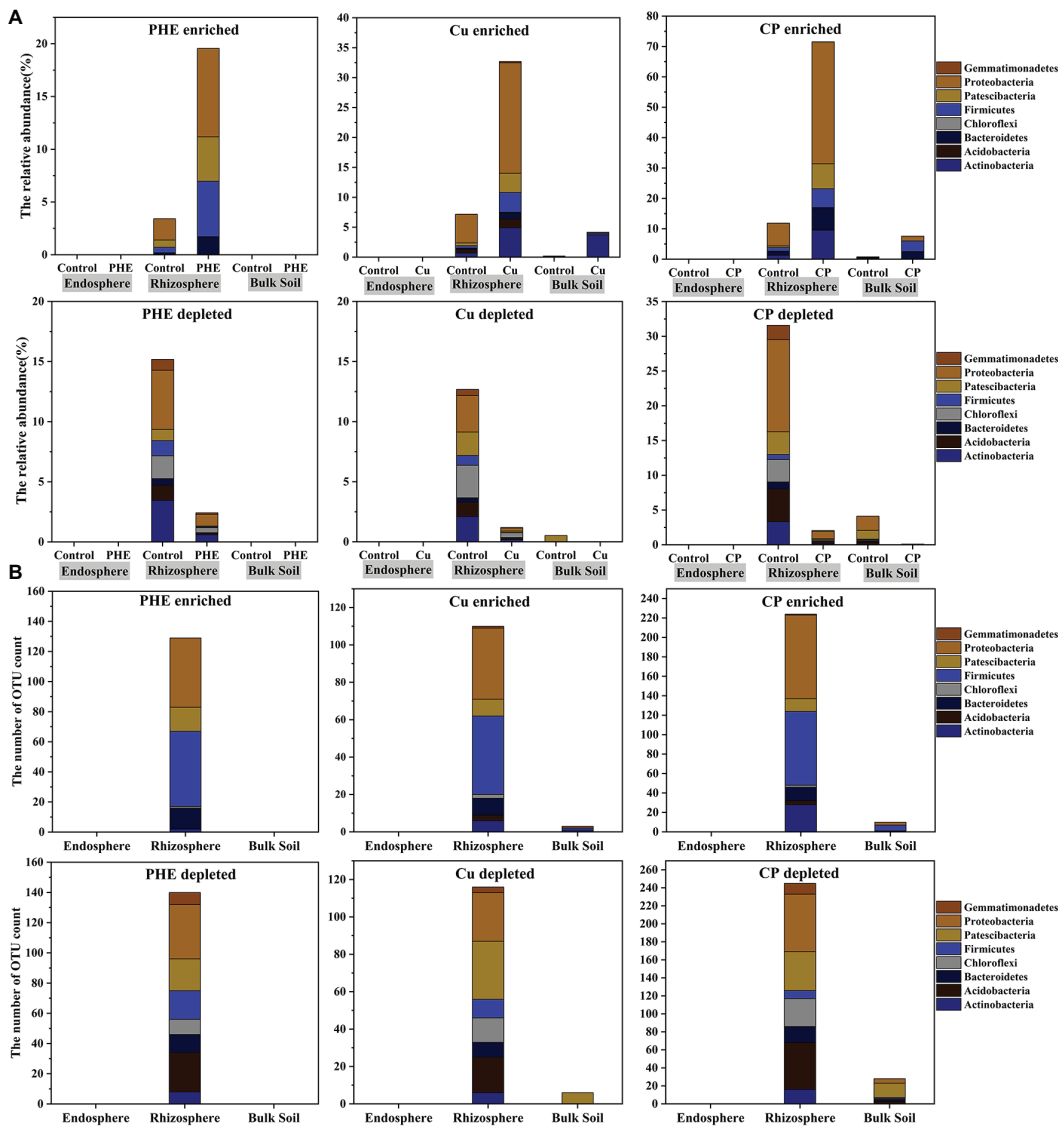
The relative abundance **(A)** and number **(B)** of enriched and depleted bacterial OTUs in different rhizocompartments under different treatments. The name abbreviation of treatment is the same as Figure 1.

To better classify the detailed root-associated microbial species that were susceptible to exogenous pollutants, the significantly enriched and depleted bacterial OTUs compared between the polluted (PHE, Cu, and PHE + Cu) treatments and the corresponding unpolluted control groups were selected (Supplementary Figures S2–S4; Supplementary Dataset S1). On balance, our results showed that the effect of the pollutants on the endophytic bacterial communities was minimal (Figure 5). Similar results were also found in a previous study on other species, such as *Miscanthus sinensis* (Sun et al., 2021). This indicates that the composition of endophytic microorganisms seemed to be more easily influenced by the plants themselves than by external environmental conditions, highlighting the importance of the plant selection effect on its colonized endophytes (Turner et al., 2013). Notably, from the perspective of the count and abundance of enriched OTUs, Cu and/or PHE pollutants significantly affected the structure of rhizosphere and bulk microbial communities, and the effect on the rhizosphere environment was greater than that on the unplanted bulk soil, which was evidenced by the highly significantly changed OTUs (Figure 5).

In the rhizosphere, compared with the unpolluted control group, the abundance of significantly enriched OTUs increased from 3.43 to 19.56% in the single PHE treatment and 7.19 to 39.70% in the single Cu treatment (Figure 5A). The changed abundance of enriched OTUs in the Cu treatment was significantly greater than that in the PHE treatment. In the treatment of combined Cu and PHE, the differentially enriched abundance reached 59%, indicating that microbial community structures in the rhizosphere were adjusted, and the combined pollutants had stronger synergetic effects on microbial growth than single pollutants (Figure 5A). Consistent with the abundance results, the number of enriched bacterial OTUs under the conditions of PHE, Cu, and combined PHE + Cu pollution was 129, 110, and 224, respectively (Figure 5B). The number of enriched OTUs increased significantly under combined pollution, which was approximate twice the number of single pollutants. However, in the bulk soil, the abundance of significantly enriched OTUs under Cu and combined pollution increased to 4.18 and 7.61%, respectively, compared to the unpolluted control. Through the comparison between rhizosphere and unplanted bulk soil, the counts and abundances of enriched OTUs in the rhizosphere were greater than those in unplanted bulk soil. This suggests that microbes in the rhizosphere were more sensitive to exogenous contaminants and that plants can respond to toxic effects by recruiting specific functional microbes in their surroundings. For instance, *H. cannabinus* selected specific root microbes that have metal-tolerant potential to survive in the rhizosphere to alleviate heavy metal stress (Chen et al., 2018). In addition, compared to single pollution, the number and richness of enriched OTUs increased significantly under the combined pollution of PHE and Cu, which was consistent with the result of the *α*-diversity variation of bacterial communities (Figure 2A). Combined with the kinetic process, we noticed that although most PHE was rapidly degraded, it still had a significant effect on the composition of the microbial communities, especially for the root-associated microbiome, and the interaction of combined PHE and Cu pollution was even stronger. This was probably due to that the microbial communities in PHE-treated soils would need some time to recover to the unpolluted state. In addition, the synergistic effects of both PAHs and heavy metals might result in a greater impact on root-associated microbial communities. A similar result has been found in Lu et al. (2013) that the biocidal effect on the soil microorganisms was significantly higher when pyrene and cadmium were applied to soil together than when they have applied alone.

Furthermore, by analyzing the enriched microbial groups in the rhizosphere, we found that in the PHE-treated rhizosphere, the enriched OTUs mainly belonged to Saccharimonadia, Cytophagales, Sphingobacteriales, Clostridiales, Alphaproteobacteria, Deltaproteobacteria, and Gammaproteobacteria (Supplementary Dataset S1). Most of the enriched microbes, e.g., Sphingobacteriales Burkholderiaceae, and Bacillales, have been reported as potential PAH degraders (Lunsmann et al., 2016; Thomas et al., 2019; Li et al., 2020). Hence, these significantly enriched microbes might be potential phenanthrene-degrading bacteria in our study. Unlike PHE, the heavy metal Cu cannot be degraded by microorganisms; hence, plants can only enhance the resistance by selecting Cu-tolerant microorganisms colonized in the rhizosphere. In this study, the significantly enriched OTUs under Cu contamination mainly belonged to Clostridiales, Alphaproteobacteria, Deltaproteobacteria, Gammaproteobacteria, Actinobacteria, and Acidobacteria (Supplementary Dataset S1). Most of them have been widely studied due to their heavy metal tolerance properties. Taken together, our results emphasized the importance of plants themselves on their survivability under unfavorable environmental conditions. Compared with single Cu or PHE treatment, the types of enriched OTUs were more abundant under the combined Cu + PHE treatment. These OTUs mainly belong to Parcubacteria, Gracilibacteria, Gemmatimonadaceae, and Ignavibacteria (Supplementary Dataset S1). The high diversity of enriched OTUs in the combined pollution treatment might be due to the toxic effect of PAHs on bacterial cells, which makes it easier for heavy metals to penetrate microbial cells and affect their functions, thus having a larger impact on the microbial communities (Maliszewska-Kordybach and Smreczak, 2003).

Similarly, we also analyzed the significantly depleted OTUs among separate treatments (Figure 5). The changed variation of depleted OTUs was similar to that of enriched OTUs. The number and abundance of depleted OTUs in the rhizosphere were significantly higher than those in the unplanted bulk soil. It was also found that the effect of combined Cu + PHE treatment on the bacterial community was dramatically stronger than that of PHE or Cu-only treatment. Even with similar counts of depleted OTUs compared to enriched OTUs in the combined polluted treatment, the total changed abundance was relatively lower. This indicated that rare bacterial groups were more sensitive to exogenous environmental pollution. The results showed that a large number of depleted OTUs were affiliated with Acidobacteria in all polluted treatments. A previous study has shown that Acidobacteria can act as plant growth-promoting bacteria and have a considerable effect on plant growth (Kielak et al., 2016). Moreover, it can produce exopolysaccharides when it attaches to the root surface of plants, which helps plant roots adhere to soil particles and protect roots. Decreased Acidobacteria may have adverse effects on plant growth. In addition, even the significantly depleted OTUs in combined polluted treatments were similar to single contamination at the class level but were more diverse at the finer taxonomic level (Supplementary Dataset S1), which confirmed the synergetic effect of combined pollution, as the results of enriched OTUs concluded.

### Significantly Changed OTUs Associated With Redox Processes

Redox processes are crucial to the balance of matter and energy cycles in ecology, and they can be intensely driven by anaerobic microorganisms in flooded paddy environments (Huang et al., 2021). Hence, we also analyzed the variation in potential rice root-associated microbes associated with redox dynamics under Cu/PHE threat conditions (Supplementary Dataset S1). Interestingly, we observed significant enrichment of iron reducer (*Geobacter*) in Cu-treated soils. *Geobacter* has not only been widely reported to have incomparable Fe (III) reduction potential (Lovley et al., 2004) but has also been confirmed to degrade many organic pollutants, e.g., benzine and phenol (Jiang et al., 2020). Similarly, some functional microbes associated with redox processes greatly decreased under polluted treatments. For example, *Desulfovibrio*, a sulfur-reducing bacterium, was significantly reduced under both PHE and combined Cu + PHE treatments. In addition, the relative abundance of *Methanobacterium*, which is associated with methane production, was significantly reduced under polluted treatments. Generally, exogenous pollution considerably influenced the functional redox microbes, which further altered the predominate cycles of biogenic elements in paddy soils.

### Commonly and Specifically Enriched and Depleted OTUs in Different Treatments

**Figure 6:**
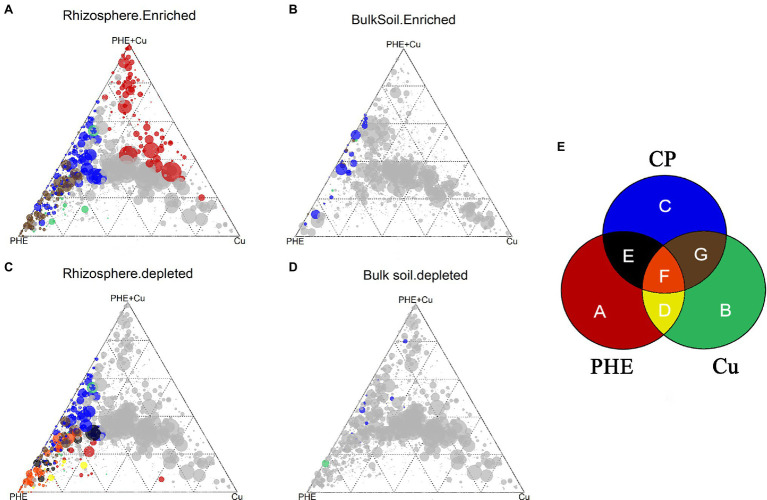
Ternary plots depicting enriched **(A**,**B)** and depleted **(C**,**D)** OTUs in the rhizosphere **(A**,**C)** and unplanted bulk soil **(B**,**D)** under different treatments. Each point corresponds to an OTU. Its size represents the average abundance across all three compartments. Colored circles represent OTUs enriched in one rhizocompartment. The sections represented by different colors are shown **(E)**. The name abbreviation of treatment is the same as Figure 1.

We also compared common and specific OTUs in different rhizocompartments under PHE-, Cu-, and combined Cu + PHE-polluted treatments to determine the similarity and difference between combined and single contamination treatments (Figure 6; Supplementary Dataset S2). In the rhizosphere, 24 OTUs belonging to Alphaproteobacteria, Gammaproteobacteria, Clostridia, and Bacteroidia were enriched in all the polluted treatments (Figure 6A). Most of these microbes have been shown to have strong tolerance or degradation potential for organic and/or inorganic pollutants (Sun et al., 2010; Zhu et al., 2020). Among these OTUs, the relative abundance of three OTUs affiliated with Candidatus Kaiserbacteria, Herbaspirillum, and Saccharimonadales varied greatly in all treatments (variation of 0.5–5.3%). *Herbaspirillum* has been considered a nitrogen-fixing bacterium with strong heavy metal tolerance (Pang et al., 2016), while Saccharimonadales also has shown significant effects on nitrogen cycling-related genes (Shi et al., 2021) and has degradation potential for PAH pollutants (Li et al., 2020). Hence, these two types of groups are suitable for use as microbial agents to remediate agricultural soil polluted by PAHs and heavy metals. The numbers of shared OTUs between combined pollution and Cu-only pollution (50 OTUs) were higher than those between combined pollution and PHE-only pollution (36 OTUs). Since most PHE has been degraded, new root microbial structures were more strongly influenced by Cu than PHE under combined pollution. Similar trends were also found in the significantly enriched OTUs in the unplanted bulk soil (Figures 6B–D), indicating that copper plays a dominant role in the interaction between PHE and Cu. Additionally, specifically enriched OTUs were also detected, and the highest number of 159 specific OTUs was found in the combined PHE + Cu-treated rhizosphere, implying that the interaction between PHE and Cu affects bacterial groups more (Supplementary Dataset S2).

### Comparison of a Root-Associated Microbial Assemblage of Flooding and Dry Farming Systems Under Heavy Metal and PAH Stress

Plants and microorganisms have various strategies to deal with heavy metal and PAH stress under several environmental conditions. We previously studied the root-associated microbial variation of dryland crop wheat in response to PHE and Cu stress (Xu et al., 2021). Hence, it is interesting to compare the similarities and differences of microbial changes between the dry-growing plant of wheat and the flooded plant of rice, as well as the response strategies of plants and root-associated microbes under contaminated soils. By comparing the dynamic process of both Cu and/or PHE pollutants, similar results were found that the indigenous microbiome in the tested agricultural soil has a strong potential to degrade PHE, and the degradation rate reached approximately 90% under both anaerobic flooding and aerobic dryland conditions. Also, considering that phenanthrene is relative a low molecular polycyclic aromatic hydrocarbon with high volatility, water solubility, and high bioavailability, which was considered to be easily degraded in the soil environment (Crampon et al., 2014). In these two processes, plants played a positive role in the degradation of PHE by increasing its availability and stimulating the growth of functional microorganisms. Both PHE and Cu were assimilated by wheat and rice roots. Cu promoted the accumulation of PHE in the interior root, but PHE had no significant effect on the enrichment of Cu. However, pyrene can decrease the accumulation of Cu in wheat with Cu-pyrene containment (Lin et al., 2008). This may indicate that oxygen conditions may be less important than other factors (e.g., the type of pollutants) in the interaction between organic matter and heavy metals.

Considering the root-associated bacterial community diversities and compositions between wheat and rice plants under polluted conditions, we found many similar and dissimilar results in the two different cropping systems. A similar variation was that in both systems, rhizocompartment (endosphere, rhizosphere, unplanted bulk soil) was identified as the main variation source of the bacterial communities. This suggested that under dry farming or flooding conditions, plants can recruit specific microbes from their surrounding soils and screen for specific microbes to enter their interior roots, resulting in significantly different bacterial community structures in separate rhizocompartments (Doornbos et al., 2012; Xu et al., 2019). The toxic stress of Cu and PHE had no significant effects on the diversity of the bacterial community in the endosphere and bulk soil, while having a strong inhibitory effect on the rhizosphere microbes. Under pollution stress, both rice and wheat plants can recruit functional- and metal-tolerant microbiota to enrich the rhizosphere, thus reducing the effects of pollution stress on the plants themselves.

In contrast, the interaction between PHE and Cu was evident in the combined pollution under the flooded environment, but not observed under dry farming conditions. Additionally, different microbes were enriched in PHE- and/or Cu-stressed environments, even with similar PHE degradation or metal tolerance potentials. This was mainly due to the difference in soil oxygen content, water content, and other conditions between dry land and flooded systems, contributing to distinct root-associated microbial composition and functionality, which lead to their unique microbial-related strategies to effectively cope with pollution stress.

## Conclusion

In this study, we found that the root-associated microbiome can be influenced by exogenous pollution stress, and the microbes with good tolerance to pollutants or degradation ability were likely to be attracted to the rhizosphere by the plant itself, thus reducing the toxic effect of pollutants on plant growth and microbes. Microbes, e.g., Sphingobacteriales Burkholderiaceae, and Bacillales, might be potential PHE-degrading bacteria enriched in the rhizosphere in our study. Due to their strong synergetic effect, it is impossible to ignore the interaction between heavy metals and PAHs on the root-associated microbial community structure and the degradation of pollutants in rice planting conditions, which is different from aerobic conditions. The degradation pathways, migration, and transformation of phenanthrene and copper were different under several oxygen conditions, which may be the reason for the variations in their interaction. Meanwhile, the synergetic effects will be due to the varieties of plants and pollutants, as well as the environmental factors. Therefore, the adaptability of microbes and the types of pollutants and plants should be carefully considered in the soil remediation of combined pollution of PAHs and heavy metals. Considering the significant differences of the bioavailability and bioaccessibility of pollutants between the artificially contaminated soils and historically contaminated soils, further studies should also be conducted to better apply the root microbial strategies to enhance the crop resistance under historically contaminated agricultural areas.

## Data Availability Statement

The datasets presented in this study can be found in online repositories. The names of the repository/repositories and accession number(s) can be found in the article/Supplementary Material.

## Author Contributions

ML: writing—review and editing, methodology, investigation, writing—original draft, and formal analysis. MX, AS, and YZ: investigation, formal analysis, and validation. NL: conceptualization, review and editing. YX: conceptualization, writing—review and editing, funding acquisition, project administration, and supervision. All authors contributed to the article and approved the submitted version.

## Funding

This research was financially supported by the Qingdao Demonstration and Guidance Project of Science and Technology to Benefit the People (21-1-4-sf-17-nsh), Natural Science Foundation of Shandong Province (ZR2019YQ18), the National Natural Science Foundation of China (51878363 and 42177028), and the Science and Technology Support Plan for Youth Innovation of Colleges in Shandong Province (DC2000000961).

## Conflict of Interest

MX was employed by Shandong Academy of Environmental Sciences Co., Ltd.

The remaining authors declare that the research was conducted in the absence of any commercial or financial relationships that could be construed as a potential conflict of interest.

## Publisher’s Note

All claims expressed in this article are solely those of the authors and do not necessarily represent those of their affiliated organizations, or those of the publisher, the editors and the reviewers. Any product that may be evaluated in this article, or claim that may be made by its manufacturer, is not guaranteed or endorsed by the publisher.

## Footnotes

^1^ https://github.com/OpenGene/fastp

^2^ https://www.arb-silva.de/

^3^ http://www.magichand.online
