# Supplementary material for: Combined Phenanthrene and Copper Pollution Imposed a Selective Pressure on the Rice Root-Associated Microbiome
Source: Front Microbiol. 2022 May 4;13:888086. doi: 10.3389/fmicb.2022.888086 (PMC9114715; doi:10.3389/fmicb.2022.888086)
Supplement: Supplementary file 1 [file Data_Sheet_1.docx]

**Supporting information**

**Table. S1.** The content of nutrient elements in the original soil.

|  | Cu | Ca | Fe | Mg | K | Ge | P | DOC |
| --- | --- | --- | --- | --- | --- | --- | --- | --- |
| Content（mg·Kg-1） | 43.46 | 3789.9 | 23598.8 | 5263.6 | 427.2 | 93.7 | 438.1 | 34.7 |

**Table. S2.** Differences in bacterial *β*-diversity between samples revealed by PERMANOVA.

| **Whole data** | | | | | | | |
| --- | --- | --- | --- | --- | --- | --- | --- |
| Factor | Df | Sums Of Sqs | Mean Sqs | F.Model | R^2^ | p value | Significance |
| Treatment | 3 | 1.423 | 0.474 | 3.228 | 0.128 | 0.001 | *** |
| Rhizocompartment | 2 | 3.954 | 1.977 | 13.450 | 0.355 | 0.001 | *** |
| Treatment: Rhizocompartment | 6 | 1.336 | 0.223 | 1.516 | 0.120 | 0.008 | ** |
| Residuals | 30 | 4.409 | 0.147 |  | 0.396 |  |  |
| Total | 41 | 11.123 |  |  | 1.000 |  |  |
| **Endosphere** | | | | | | | |
|  |  |  |  |  |  |  |  |
| Factor | Df | Sums Of Sqs | MeanSqs | F.Model | R^2^ | p value | Significance |
| Treatment | 3 | 1.078 | 0.359 | 1.875 | 0.338 | 0.001 | *** |
| Residuals | 11 | 2.107 | 0.192 |  | 0.662 |  |  |
| Total | 14 | 3.185 | 0.000 |  | 1.000 |  |  |
| **Rhizosphere** | | | | | | | |
| Factor | Df | Sums Of Sqs | MeanSqs | F.Model | R^2^ | p value | Significance |
| Treatment | 3 | 1.021 | 0.340 | 3.866 | 0.513 | 0.001 | *** |
| Residuals | 11 | 0.969 | 0.088 |  | 0.487 |  |  |
| Total | 14 | 1.990 |  |  |  |  |  |
| **Bulk soil** | | | | | | | |
|  |  |  |  |  |  |  |  |
| Factor | Df | Sums Of Sqs | MeanSqs | F.Model | R^2^ | p value | Significance |
| Treatment | 3 | 0.673 | 0.224 | 1.345 | 0.335 | 0.032 | * |
| Residuals | 8 | 1.333 | 0.167 |  | 0.665 |  |  |
| Total | 11 | 2.006 |  |  | 1.000 |  |  |

**Table.S3.** Differences in archaeal *β*-diversity between samples revealed by PERMANOVA.

| **Whole data** | | | | | | | |
| --- | --- | --- | --- | --- | --- | --- | --- |
| Factor | Df | Sums Of Sqs | Mean Sqs | F.Model | R^2^ | *p* value | Significance |
| Treatment | 3 | 0.731 | 0.244 | 1.366 | 0.640 | 0.130 |  |
| Rhizocompartment | 2 | 4.256 | 2.128 | 11.933 | 0.373 | 0.001 | *** |
| Treatment: Rhizocompartment | 6 | 1.080 | 0.180 | 1.009 | 0.095 | 0.430 |  |
| Residuals | 30 | 5.350 | 0.178 |  | 0.469 |  |  |
| Total | 41 | 11.418 |  |  | 1.000 |  |  |
| **Endosphere** | | | | | | | |
| Factor | Df | Sums Of Sqs | Mean Sqs | F.Model | R^2^ | *p* value | Significance |
| Treatment | 3 | 1.195 | 0.398 | 0.955 | 0.261 | 0.558 |  |
| Residuals | 11 | 4.591 | 0.417 |  | 0.793 |  |  |
| Total | 14 | 5.786 |  |  | 1.000 |  |  |
| **Rhizosphere** | | | | | | | |
| Factor | Df | Sums Of Sqs | Mean Sqs | F.Model | R^2^ | *p* value | Significance |
| Treatment | 3 | 0.452 | 0.151 | 3.545 | 0.492 | 0.004 | ** |
| Residuals | 11 | 0.467 | 0.042 |  | 0.508 |  |  |
| Total | 14 | 0.919 |  |  | 1.000 |  |  |
| **Bulk soil** | | | | | | | |
| Factor | Df | Sums Of Sqs | Mean Sqs | F.Model | R^2^ | *p* value | Significance |
| Treatment | 3 | 0.141 | 0.047 | 1.286 | 0.325 | 0.135 |  |
| Residuals | 8 | 0.293 | 0.037 |  | 0.675 |  |  |
| Total | 11 | 0.434 |  |  | 1.000 |  |  |


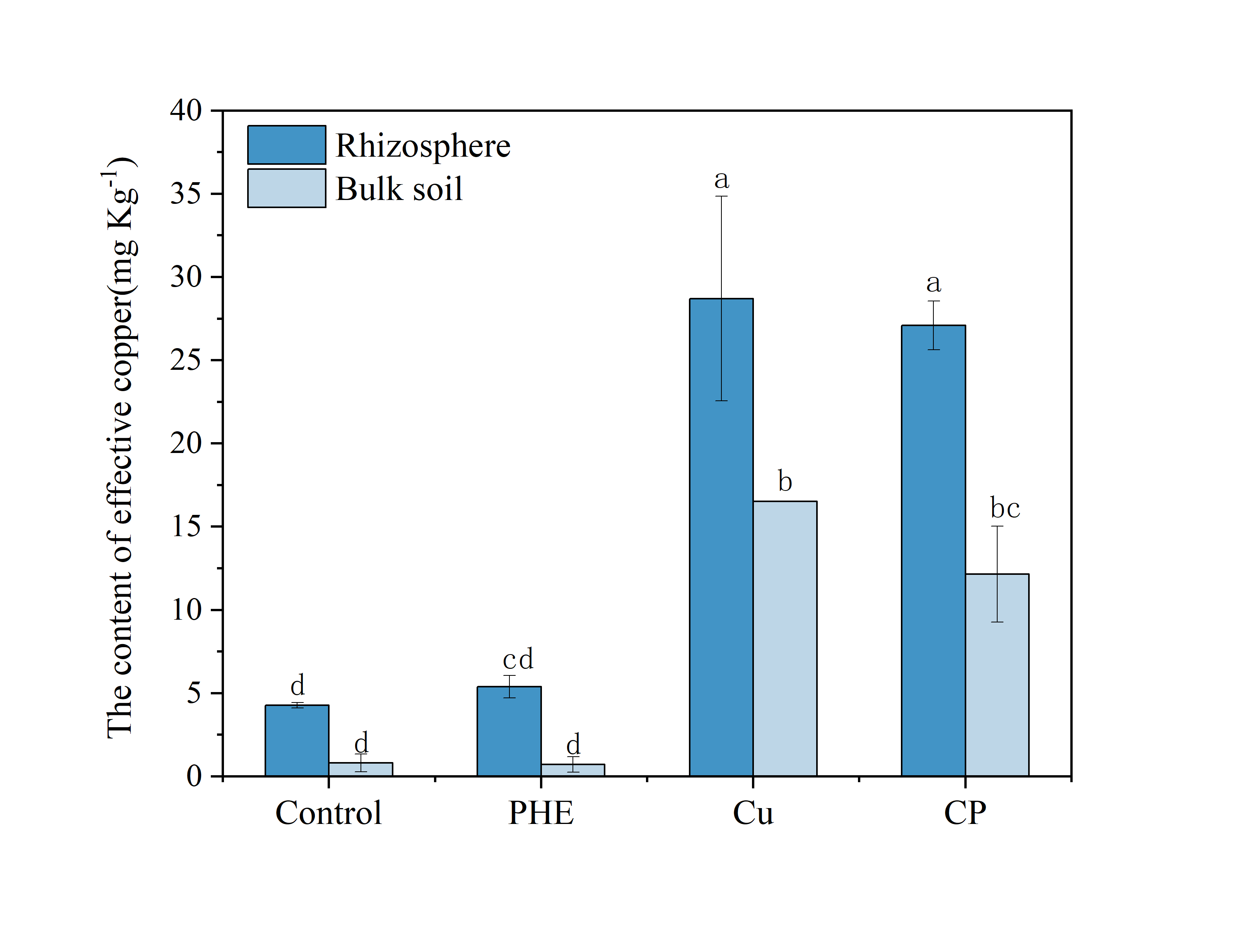


**Fig. S1.** The concentrations of effective copper in the rhizosphere and unplanted bulk soil. Symbol meaning: PHE, phenanthrene-only treatment; Cu, Cu-only treatment; CP: composite treatment of phenanthrene and Cu.

**
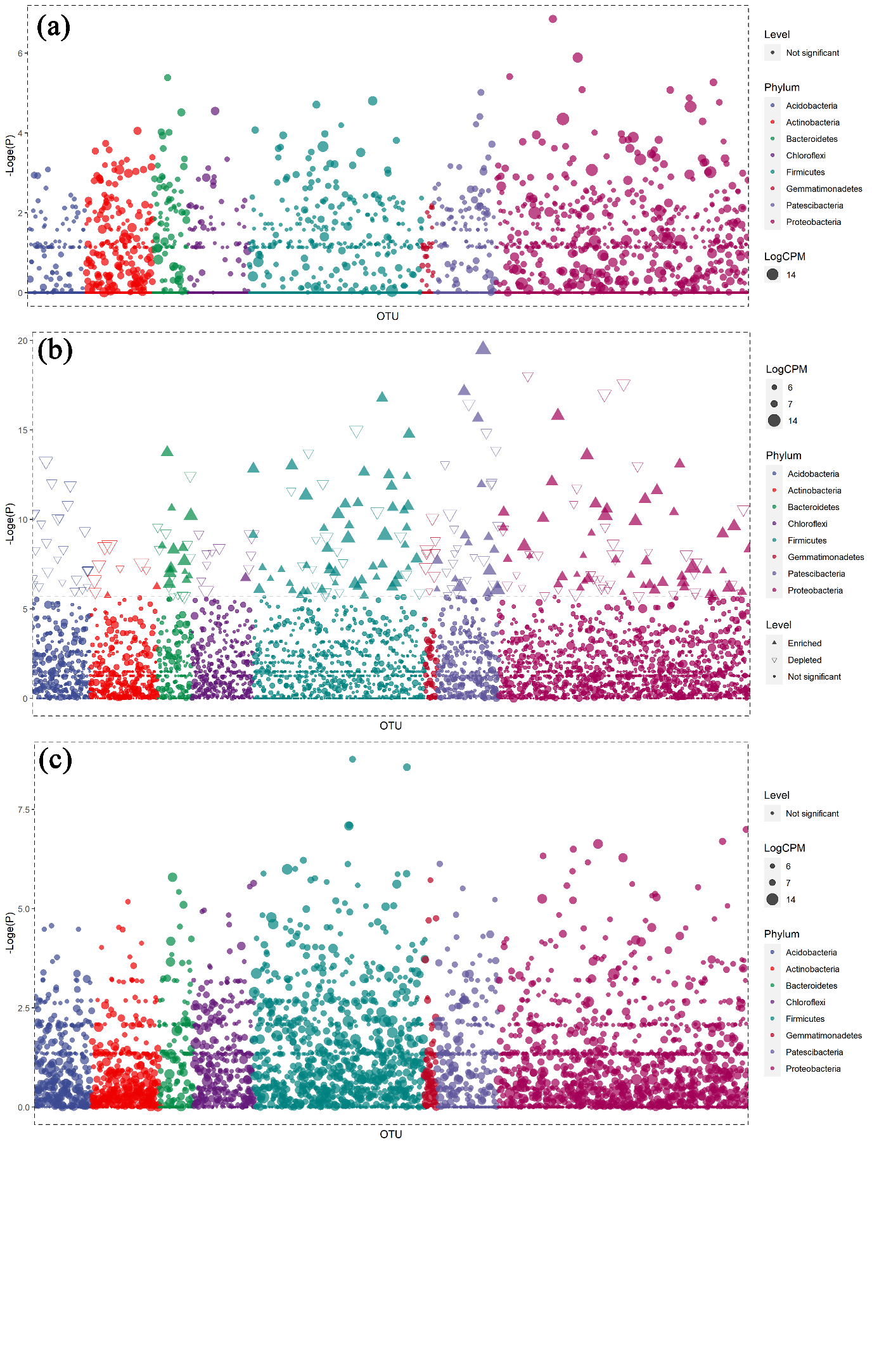
Fig. S2.** Enriched and depleted bacterial OTUs in PHE treated endosphere (a), rhizosphere (b) and bulk soil (c).

**
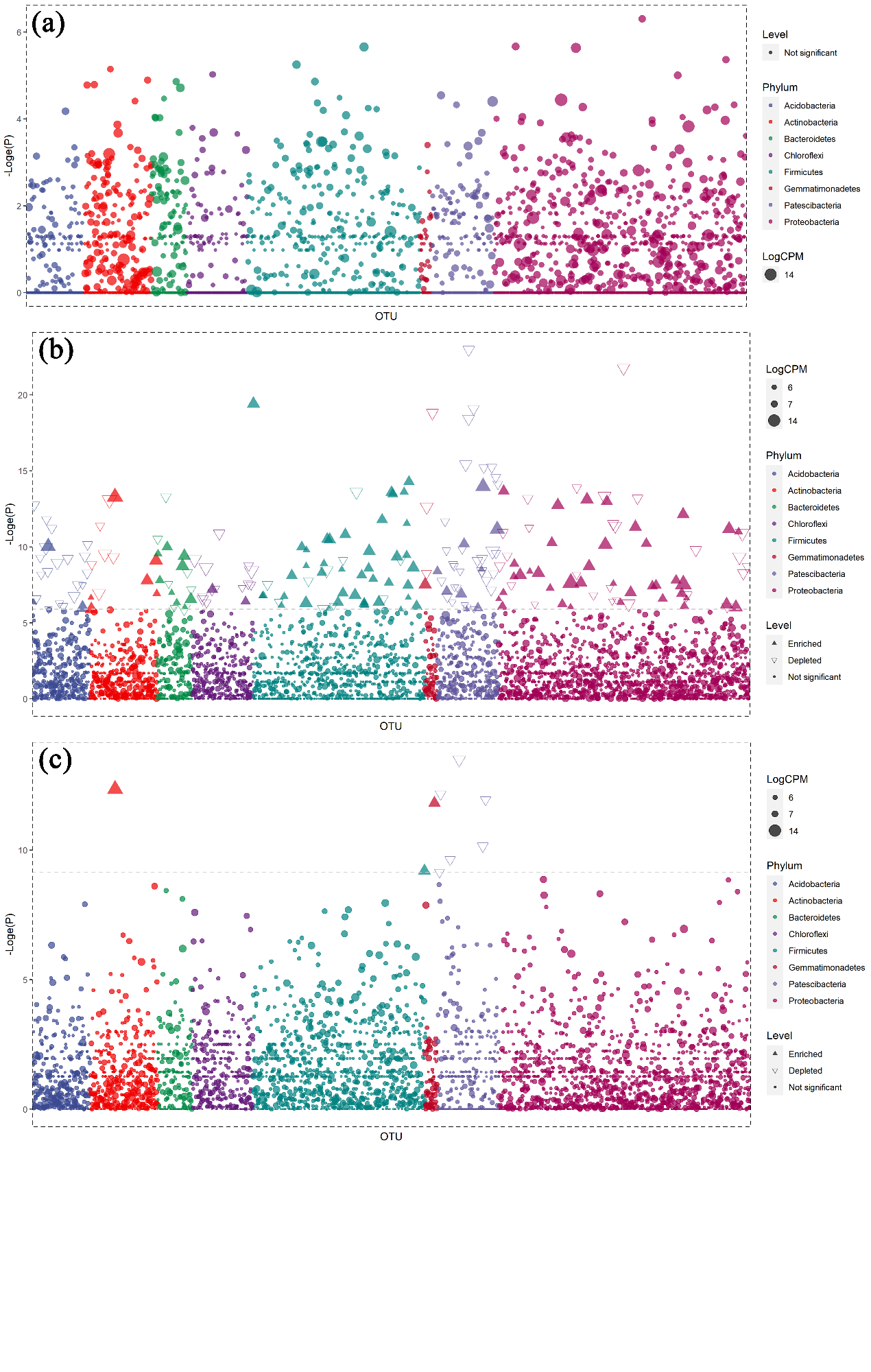
Fig. S3.** Enriched and depleted bacterial OTUs in Cu treated endosphere (a), rhizosphere (b) and bulk soil (c).

**
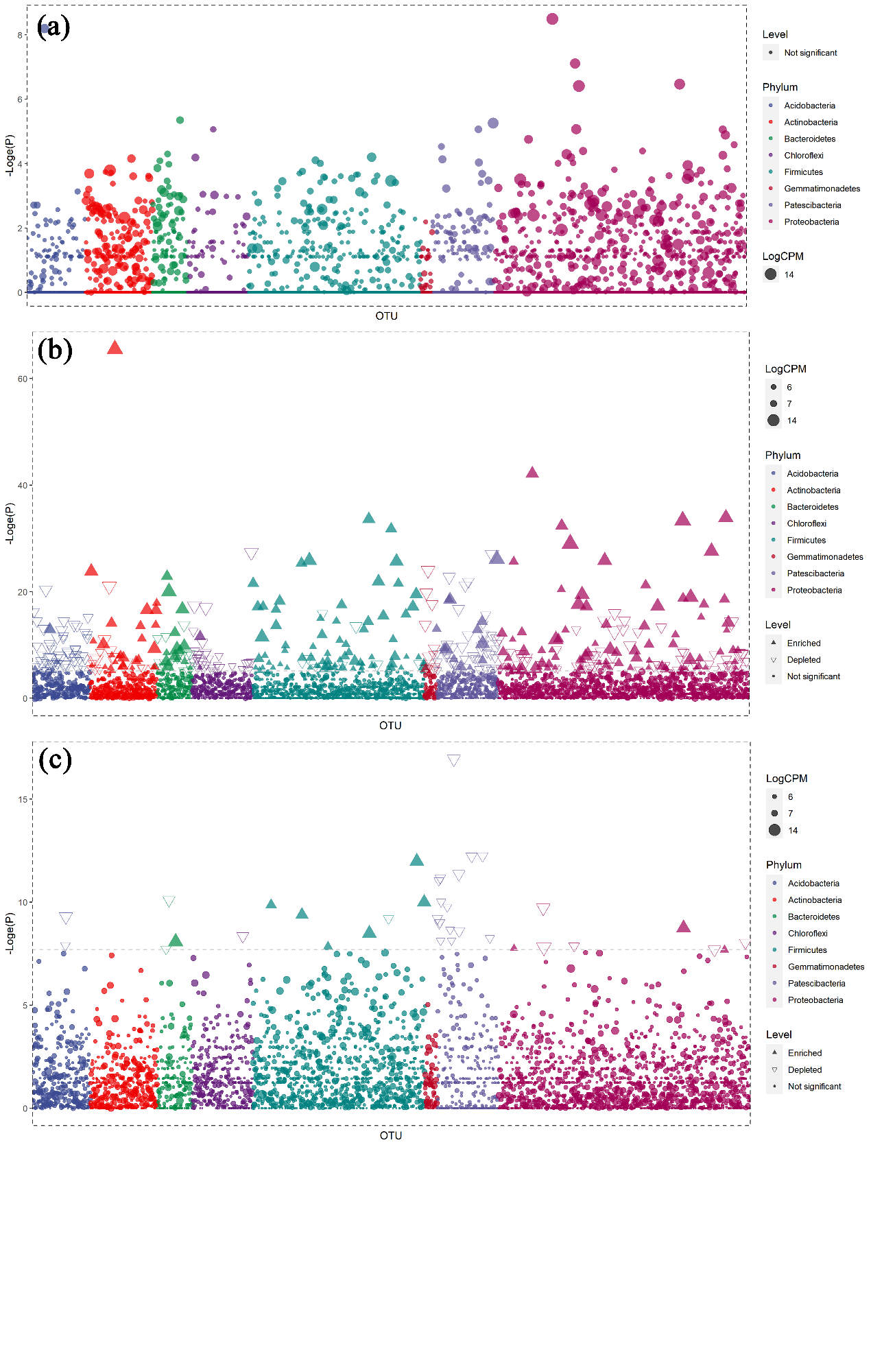
Fig. S4.** Enriched and depleted bacterial OTUs in Cu+PHE treated endosphere (a), rhizosphere (b) and bulk soil (c).

**b**
